# A national research centre for the evaluation and implementation of person‐centred care: Content from the first interventional studies

**DOI:** 10.1111/hex.13120

**Published:** 2020-08-18

**Authors:** Hanna Gyllensten, Ida Björkman, Eva Jakobsson Ung, Inger Ekman, Sofie Jakobsson

**Affiliations:** ^1^ Centre for Person‐Centred Care (GPCC) University of Gothenburg Gothenburg Sweden; ^2^ Institute of Health and Care Sciences Sahlgrenska Academy University of Gothenburg Gothenburg Sweden

**Keywords:** clinical trials, interdisciplinary research, patient‐centred care, person‐centred care, person‐centred care, surveys and questionnaires

## Abstract

**Background:**

Person‐centred care (PCC) has been suggested as a potential means to improve the care of patients with chronic and long‐term disorders. In this regard, a model for PCC was developed by the University of Gothenburg Centre for Person‐Centred Care (GPCC).

**Objective:**

The present study aimed to explore the theoretical frameworks, designs, contexts and intervention characteristics in the first 27 interventional studies conducted based on the ethics for person‐centredness provided by the GPCC.

**Design:**

Cross‐sectional study.

**Setting and participants:**

A questionnaire to the principal investigators of the 27 intervention studies financed by the GPCC and conducted between 2010 and 2016.

**Main outcome measures:**

Theoretical frameworks, contexts of studies, person‐centred ethic, and outcome measures.

**Results:**

Most of the interventions were based on the same ethical assumptions for person‐centredness but theories and models in applying the interventions differed. All studies were controlled; 12 randomized and 15 quasi‐experimental. Hospital in‐ and outpatient and primary care settings were represented and the outcome measures were related to the specific theories used. A complexity in designing, introducing and evaluating PCC interventions was evident.

**Conclusion:**

The frameworks, designs and interventions in the studies were in line with the established ethical basis of PCC, whereas outcome measures varied widely. Consensus discussions among researchers in the field are needed to make comparisons between studies feasible.

**Patient or public contributions:**

Patients or the public made no direct contributions, although most of the studied projects included such initiatives.

## 1 INTRODUCTION

Healthcare systems need to be re‐organized to provide high‐quality care without increased costs to an ageing population with a high prevalence of chronic and long‐term disorders.^1^ Many western countries face numerous challenges in which the demand for health care is expected to steadily increase because of demographic and epidemiological changes.^2^ Swedish health care, compared with other countries, performs relatively well regarding medical care.^1^ However, even in Sweden long waiting times for care, health inequities based on socioeconomic factors and poor care coordination and lack of effective care models are all pressing issues.^1^, ^3^ In addition, continuity, availability, patient involvement and satisfaction with care are less than optimal.^3^, ^4^ According to some, the healthcare system needs to decrease costs and improve care quality.^5^ Different solutions have been proposed to acknowledge the patient in health care, including patient‐centred and person‐centred care (PCC) initiatives. While both can be seen as alternatives to a more paternalistic biomedical paradigm, patient‐centred care has been described as being more oriented to functioning and PCC as more directed to a meaningful life.^6^ The University of Gothenburg Centre for Person‐Centred Care (GPCC) (www.gpcc.gu.se) was established in February 2010 and formalized as the first centre in Europe to enhance and coordinate interdisciplinary research in PCC.^7^

PCC is based on a philosophical approach to acknowledge and endorse the individual's resources, interests, needs and preferences. From a PCC perspective, healthcare professionals see patients as partners in the planning and performing of the care process. Moreover, PCC comprises shared responsibility, coordinated care and treatment.^8^, ^9^, ^10^ In a previously published logic model for PCC, developed for the American Geriatrics Society, emphasis is also put on involving other family members in the care.^11^

Early research has shown that an intervention based on PCC after surgery was successful in enhancing activities of daily living, improving care satisfaction and reducing hospital admissions.^12^ Based on these findings, Ekman et al^9^ illustrated how the ethics of person‐centredness could be operationalized in practice through PCC, in which the theoretical framework encompasses the philosophy of personhood manifested through the patient narrative, partnership and coherent documentation,^9^ often called the three cornerstones of PCC. One of the first controlled studies based on this framework—‘the Gothenburg model of PCC’ (hereafter referred to as the gPCC, not to be confused with ‘GPCC’, the research centre itself)—showed, in line with the findings of Olsson et al,^12^ reduced hospital stay for patients with chronic heart failure without worsening functional performance or increasing the risk of readmission.^13^

Previous evaluations have reported on how health professionals translate the gPCC to their clinical practice^14^ and in what way involved participants understand the partnership created when using this model.^15^ In these studies, healthcare professionals had to interpret how to apply the gPCC in their setting,^14^ and that there are aspects of the partnership created through PCC not directly linked to what is written in the health plan.^15^ However, less is known about whether uniformity exists as to how the gPCC and its intended effects have been operationalized and evaluated. A PCC intervention is a complex and challenging objective in that it contains several interacting components.^16^, ^17^ For example, the elements included in the interventions should be tailored to each participant and different clinical contexts for which the potential outcomes can be multiple and dispersed rather than linear. The design and evaluation of complex interventions need to be handled in relation to the complexity involved,^16^ including understanding how the interventions are produced and affect participants and the settings in which they are tested and later implemented.

The present study aimed to explore the theoretical frameworks, designs, contexts, intervention characteristics and outcome measures in the first 27 interventional studies conducted based on the ethics for person‐centredness provided by the GPCC.

## 2 METHODS

A questionnaire was developed to explore methodological aspects concerning design and evaluation in the 27 interventional studies. The questionnaire (Appendix A) contains questions on how the intervention was person‐centred,^9^ the development of the intervention (including any pilot studies conducted),^18^ the intervention itself (study population, etc),^19^ evaluation and outcome measures (including adverse outcomes),^20^, ^21^ implementation measures,^22^ the current status of the study and eventual publications. Items included in the questionnaire were constructed to be consistent with recognized reporting standards and guidelines (including the TIDieR (Template for Intervention Description and Replication checklist),^19^ Medical Research Council, developing and evaluating complex interventions,^23^ Criteria for Reporting the Development and Evaluation of Complex Interventions in healthcare: revised guideline (CReDECI 2)^18^ and Consolidated Standards of Reporting Trials (CONSORT).^20^ The questionnaire was piloted and discussed with the GPCC steering committee. In May 2016, the questionnaire was sent to the principal investigators (PIs) of the 27 interventional studies financed by GPCC and conducted in 2010‐2016. Those PIs not responding initially were reminded during the autumn of 2016.

Frequencies were used to analyse the close‐ended questions. Categories, either based on inductive or deductive analysis, were developed based on the open‐ended questions. The inductive analysis sought to describe the content and operationalization of the intervention as regards the philosophy of PCC.^9^ The deductive categories for analysing and reporting outcome measures were based on the ECHO model (for Economic, Clinical and Humanistic Outcomes), costs and economic outcomes, clinical intermediaries and outcomes (measured by professionals) and humanistic intermediaries and outcomes (self‐reported by patients/users).^24^ Outcome measures were also assessed for their ability to represent the various aspects of PCC, as described by De Silva.^25^ Categories were discussed in different forums (such as open workshops for researchers associated with GPCC and steering committee meetings) during the analysis process.

## 3 RESULTS

All PIs responded before February 2017 (100% response rate). Between 2010 and 2016, 27 studies (12 randomized controlled trials and 15 quasi‐experimental) were financed and performed within the centre (Table 1). Of the 27 studies, 12 were described as multi‐centre studies. Most of the studies reported that the study interventions were designed and adjusted relative to the different study populations by the investigators and in 19 studies, this was also done in collaboration with clinicians. Eight of the studies reported that other research groups had been consulted and in 14 studies, external expertise and patient representatives collaborated in the design and adjustment of the intervention. When the PIs responded to the questionnaire (2016‐2017), 12 of the projects were completed and 18 had resulted in peer‐reviewed original articles.

**TABLE 1:** Description of person‐centred care interventional studies in this paper

Study	Project title	Study design	Context	Intervention provider/s	Study population*	Sample size	Theoretical perspective/s	PCC cornerstones^†^
a	Evaluation of training and supervision in supported communication for medical students.	Quasi‐experimental, non‐randomized controlled trial	Medical school	Speech therapists	Medical students	≤50	Person‐centredness, Interaction and communication, learning, self‐efficacy	Patient narrative Partnership
b	Person‐centred information and communication technology support to people with chronic heart failure, and/or COPD	Experimental, randomized controlled trial	Hospital‐based outpatient care	Nurses	Patients with chronic heart failure, and/or COPD	51‐150	Person‐centredness, self‐efficacy, health, symptoms, coping and profession‐specific	Patient narrative Partnership Documentation
c	Person‐centred care after acute coronary syndrome	Experimental, randomized controlled trial, multi‐centre	Hospital‐based inpatient and outpatient care and primary care	Physicians, Nurses	Patients with acute coronary syndrome	151‐250	Person‐centredness, self‐efficacy, health, symptoms, coping and profession‐specific	Patient narrative Partnership Documentation
d	Effects of person‐centred care in patients with chronic heart failure	Quasi‐experimental, non‐randomized controlled trial	Hospital‐based inpatient care	All healthcare professionals at the ward	Patients with chronic heart failure	151‐250	Person‐centredness, self‐efficacy, health, symptoms, coping and profession‐specific	Patient narrative Partnership Documentation
e	Evaluation of a training programme to facilitate communication between adult persons with communication disorders and their relatives	Quasi‐experimental, non‐randomized controlled trial	Community‐based networks/services	Speech therapists	Residents with communication disorders and their relatives	≤50	Interaction and communication, learning	Patient narrative Partnership
f	Evaluation of a training programme to facilitate communication between adult persons with communication disorders and nurse assistants	Quasi‐experimental, non‐randomized controlled trial, multi‐centre	Community‐based residential care facility	Speech therapists	Residents with communication disorders and nurse assistants	≤50	Person‐centredness, interaction and communication, learning	Patient narrative Partnership
g	Evaluation of person‐centred communication in nursing homes	Quasi‐experimental, before/after study multi‐centre	Community‐based residential care facility	Speech therapists	Nurse assistants	51‐150	Person‐centredness, interaction and communication	Patient narrative Partnership Documentation
h	Effects of an implementation of a person‐centred approach on older person´s quality of life and incontinence care at residential care facilities	Quasi‐experimental, before/after study, multi‐centre	Community‐based residential care facility	All healthcare professionals at the resident	Residents and caregivers	51‐150	Person‐centredness	Patient narrative Partnership Documentation
i	Person‐centred support for persons with irritable bowel syndrome	Quasi‐experimental, before/after study	Hospital‐based outpatient care	Nurse	Patients with irritable bowel syndrome	≤50	Person‐centredness, Interaction and communication, self‐efficacy, health, symptoms, coping and profession‐specific.	Patient narrative Partnership Documentation
j	Person‐centred care and the importance of the multidisciplinary cancer team for patients with head and neck cancer	Experimental, randomized controlled trial	Hospital‐based outpatient care	Physicians, Nurses	Patients with head and neck cancer	51‐150	Person‐centredness, organization and leadership	Patient narrative Partnership Documentation
k	Home‐based person‐centred care after stroke	Experimental, randomized controlled trial	Hospital‐based inpatient and outpatient care	Occupational therapists, Physiotherapists, Nurses	Patients with stroke	51‐150	Person‐centredness, health, symptoms, coping and profession‐specific, own theory development	Patient narrative Partnership Documentation
l	Person‐centred physiotherapy in major depression	Experimental, randomized controlled trial	Primary care	Physiotherapists	Patients with depression	51‐150	Person‐centredness	Patient narrative Partnership Documentation
m	Help overcoming pain early: an evaluation of person‐centred support for adolescents	Experimental, randomized controlled trial,multi‐centre	School health service	Nurse specialists	Adolescents with chronic pain	51‐150	Person‐centredness,health, symptoms, coping and profession‐specific, own theory development	Patient narrative Partnership Documentation
n	Person‐centred health promotion to support capability persons 70 + who have migrated to Sweden	Experimental, randomized controlled trial	Community‐based networks/services	Social Workers,Occupational therapists,Physiotherapists,Nurses	Persons born abroad	51‐150	Health, symptoms, coping and profession‐specific	Patient narrative Partnership Documentation
o	Safe Hands at the Sharp End: implementing aseptic technique in the care of frail persons undergoing acute hip surgery	Quasi‐experimental, non‐randomized controlled trial	Hospital‐based inpatient care	Surgical teams	Surgical teams		Person‐centredness,Organization and leadership	Patient narrative Partnership Documentation
p	Evaluation of person‐centred care at an internal medicine ward	Quasi‐experimental, before/after study	Hospital‐based inpatient care	All healthcare professionals at the ward	Patients admitted to an internal medicine ward	>250	Person‐centredness	Patient narrative Partnership Documentation
q	Person‐centred web‐based support for women with type 1 diabetes during pregnancy and early motherhood	Experimental, randomized controlled trial,multi‐centre	Hospital‐based outpatient care	Peers	Women with type 1 diabetes during pregnancy	151‐250	Person‐centredness,own theory development.	Patient narrative Partnership
r	Person‐centred information and communication in partnership: a stepwise intervention for patients undergoing colorectal cancer surgery	Quasi‐experimental, before/after study,multi‐centre	Hospital‐based inpatient and outpatient care	Physicians,Nurse	Patients with colorectal cancer	>250	Person‐centredness,own theory development	Patient narrative Partnership Documentation
s	Person‐centred care and rehabilitation after acute vertebral compression fracture	Quasi‐experimental, before/after study, level of caregiver,multi‐centre	Hospital‐based inpatient care	All health care professionals at the ward	Patients with acute vertebral compression fracture	>250	Person‐centredness,health, symptoms, coping and profession‐specific	Patient narrative Partnership Documentation
t	Person‐centred psychosis care	Quasi‐experimental, before/after study	Hospital‐based inpatient care	Social Workers,Physicians,Nurses, Nurse assistants	Patients with psychosis	51‐150	Person‐centredness	Patient narrative Partnership Documentation
u	Mighty Mums ‐ person‐centred care for pregnant women with BMI > 30	Quasi‐experimental, non‐randomized controlled trial,multi‐centre	Primary care	Midwives, DieticiansPhysiotherapists	Women with BMI > 30 during pregnancy	>250	Own theory development	Patient narrative Partnership Documentation
v	Person‐centred web‐based support for children with urinary bladder dysfunction.	Quasi‐experimental, non‐randomized controlled trial	Hospital‐based outpatient care	Nurse and specialist teacher	Children with urinary bladder dysfunction	≤50	Person‐centredness,health, symptoms, coping and profession‐specific	Patient narrative Partnership
w	Resistant exercise within a person‐centred care perspective	Experimental, randomized controlled trial,multi‐centre	Primary care	Physiotherapists	Patients with fibromyalgia	51‐150	Health, symptoms, coping and profession‐specific	Patient narrative Partnership Documentation
x	Mastery and autonomy in medication with a mobile phone self‐report system	Quasi‐experimental, before/after studymulti‐centre	Primary care	Physicians,Nurses	Patients with hypertension	≤50	Person‐centredness	Patient narrative Partnership Documentation
y	Mindfulness‐Based Stress Reduction: effects on symptoms and signs, perceptions of health and wellbeing in persons with chronic heart failure	Experimental, randomized controlled trial	Hospital‐based outpatient care	Nurse	Patients with chronic heart failure	≤50	Person‐centredness,own theory development	Patient narrative Partnership Documentation
s	Effects of person‐centred physical therapy on fatigue‐related variables in persons with rheumatoid arthritis	Experimental, randomized controlled trial	Hospital‐based outpatient care	Physiotherapist	Patients with rheumatoid arthritis	51‐150	Person‐centredness,health, symptoms, coping and profession‐specific	Patient narrative Partnership Documentation
aa	Evaluating a computer‐based educational programme for women diagnosed with early‐stage breast cancer	Experimental, randomized controlled trial,multi‐centre	Hospital‐based outpatient care	Not applicable	Patients with early‐stage breast cancer	151‐250	Learning	Patient narrative Partnership Documentation

Note

PCC, person‐centred care. COPD = chronic obstructive pulmonary disease.

a ^*^ Study population refers to the population that was used to evaluate the primary outcome.

b ^†^ Specified in Ekman et al 2011.^9^

### 3.1 Theoretical frameworks

Of the 27 studies, 22 reported person‐centred ethics as the conceptual framework (Table 1). Other conceptual frameworks were self‐efficacy (*n *= 5), interaction and communication theories (*n* = 5), theories on learning (*n* = 3) and theories on organization and leadership (*n* = 2). Varying definitions of health, symptoms and coping were reported as the theoretical framework in 11 studies. Such definitions could be either profession‐specific (eg nursing and occupational therapy) or generic. Six studies reported that the intervention was based on previous theory development, for example, through qualitative studies within the research group.

### 3.2 Contexts

Most of the interventions took place within hospital‐based care (inpatient or specialized outpatient care, *n* = 16). One study included several care levels: hospital‐based in‐ and outpatient care and primary care. The interventions within primary health care (*n* = 5) included maternal health services, general practitioners’ services and rehabilitation centres. Five of the intervention studies were performed within community‐based care (including municipal care) and one within a medical training school. In most of the studies, educational activities covering the theoretical framework of person‐centredness were completed by the providers of the intervention before initiation of the intervention. Educational activities (eg workshops, discussions, lectures and supervision) were conducted to facilitate the implementation of the PCC model.

Of the 27 interventions, 23 were aimed directly towards the study population while four sought to facilitate healthcare professionals’ implementation of PCC in daily practice (Table 1). The study populations included 25 interventions for adults, one for children and one for adolescents. Most studies (*n* = 22) included or excluded participants based on diagnosis, current health status and ability to participate (based on physical, cognitive or technical requirements to perform the intervention). In 13 studies, sex, age and country of birth were required characteristics of the study participants. Four studies were conducted to facilitate implementation, based their inclusion on all employees or students at the specific study site. Seventeen studies were preceded by a formalized sample size calculation or with a large study population (including controls) to enable statistical inference.

### 3.3 Operationalization of person‐centred ethics

**FIGURE 1:**
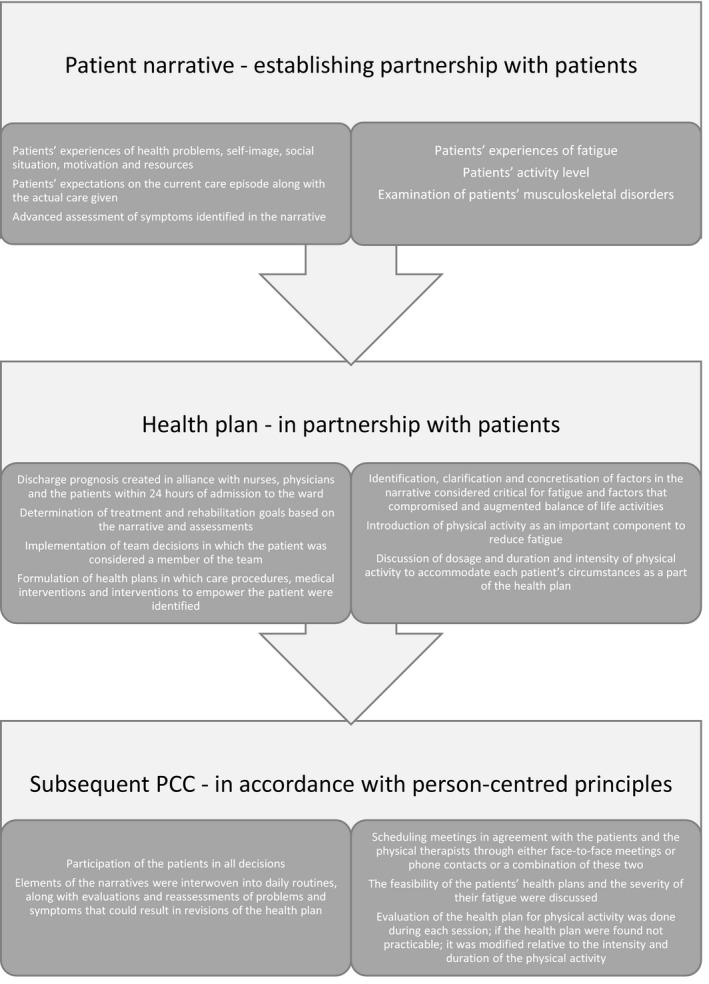
Similarities and differences in a generic vs. a specific person‐centred care interventional study. Modified descriptions of interventions evaluated in study p^42^ and z^51^ in Table 1. PCC, person‐centred care

At least two and sometimes, all three of the gPCC cornerstones of PCC (patient narrative, partnership and documentation) framed the interventions (Table 1). Half of the interventions focused on a specific health problem (eg communication disorders, incontinence, obesity and pain), with specific interventions such as communication tools and physical exercise. In contrast, the other half had a broad generic approach to problems associated with the patient's health status. Examples of the two types of intervention are described in Figure 1.

### 3.4 The content in relation to person‐centredness

The qualitative analysis, which aspired to analyse the interventions concerning the ethics of person‐centredness, revealed consistent descriptions of personhood, narration and partnership; documentation, on the other hand, was described in different terms. *Personhood* was evident as the PCC interventions had to be adapted to the unique needs, values, strengths, challenges and resources of the individual. In addition, the interventions needed to be continuously modified to changes over time and fit into the daily life of the individual. One or multiple opportunities for *the patient narrative* were evident aspects of the interventions. *The partnership* was manifested through individual meetings or peer support with other patients (contrarily, next‐of‐kin was only mentioned in a few of the interventions). The interventions also contained strategies on how to support each person over time in collaboration with the care provider. *Documentation* was described as either patient‐held documentation or shared documentation between the patient and the healthcare professional. Different plans (eg health and exercise plans) were frequently reported examples of documentation used in the PCC interventions and various health, symptom, activity and exercise diaries. Other frequent documentation materials used in the interventions included written information and educational materials for the patients. There was also documentation explicit to healthcare professionals. This explicit documentation was educational or served to be supportive in the operationalization of the intervention. It was also reported that the interventions had to be adapted and modified to evidence‐based practice, patient safety and available resources.

### 3.5 Face‐to‐face or at a distance?

All interventions but four entailed a face‐to‐face intervention (*n* = 23) (Table 2). Moreover, four studies evaluated remote telephone‐ or web‐based support additional to standard care (Table 2). The number of encounters between the patients and care providers varied from a single encounter to repeated encounters over a predesignated period. Several interventions consisted of remote support in addition to face‐to‐face contacts. The studies entailed a variety of PCC interventions (Table 1).

**TABLE 2:** Delivery of the interventions to the study population

How delivered	Contacts with intervention provider	Time frame	Number of studies*
Individually face‐to‐face	During inpatient care + structured encounters in outpatient and primary care	During and after inpatient care	1^c^
	During inpatient care	During inpatient care	3^d,p,t^
	Structured encounters	1 month	1^k^
Individually face‐to‐face + remote	During inpatient care + structured encounters in outpatient care + telephone support	During and after inpatient care	3^j,r,s^
	Structured encounters + web‐based or telephone support	2‐3 months	4^i, l, m, z^
	Structured encounters + self‐management support system + telephone support	2 months	1^x^
	Structured encounters + telephone support	~6 months	1^u^
Individually face‐to‐face or in group sessions	Structured encounters	1‐3 months	4^e, f, n, y^
	Structured encounters	4 months	1^w^
In group sessions face‐to‐face	1 encounter	‐	1^a^
	During the implementation phase	1.5 −10 months	3^g,h,o^
Remote	Telephone support one or more times + eHealth platform	6 months	1^b^
	Web‐based support	~12 months	1^q^
	Web‐based support	6 months	1^v^
	CD		1^aa^

* For study reference letters, see Table 1. Based on responses to the questionnaire items and not from the original protocol of the intervention. Responses differed in detail when describing the intervention. Telephone support = scheduled support or when needed. Encounters = consultations, physical exercise or information.

### 3.6 Several health professionals represented

Of the 27 studies, 14 reported the provision of 2‐7 health professions (the remaining interventions (*n* = 13) were provided by one profession, either a registered nurse (RN), physical therapist, speech therapist or a midwife).

#### 3.6.1 Outcome measures

In total, 163 outcome measures (specific questionnaires, health measures or other outcomes), ranging from 1 to 17 measurements per study, were reported (Table 3). Economic dimensions were covered in the evaluation of 8 studies, clinical dimensions (this means that the specific outcomes were clinician‐reported) in 14 studies and humanistic dimensions (ie self‐reported by the patients/users) in 20 studies. Six studies covered all three dimensions (economic, clinical, and humanistic) in the evaluation, and eight covered two dimensions (all of these included the humanistic dimension, together with either the economic or clinical dimension). One study based the assessment on only the economic dimension, one on only the clinical dimension and six on only the humanistic dimension. Thirteen studies included treatment modifiers (eg outcomes relating to how the intervention operated in practice), together with other variables in the evaluation (5 of these 13 studies only covered treatment modifiers). In addition, four studies reported unintended outcomes (ie the effects of an intervention other than those they sought to achieve). Nineteen of the studies included a qualitative evaluation of the intervention, mainly through interviewing patients, healthcare providers or other relevant stakeholders, but sometimes through observations or a review of medical records.

**TABLE 3:** Outcomes measured in the PCC interventions

Main category	Sub‐category	Measured dimensions	Number of studies*
Economic	Costs^†^	Direct healthcare costs	2^b, n^
	Economic outcomes^†^	Cost‐effectiveness/Cost‐utility^‡^	6^c, d, j, k, s, u^
Clinical	Intermediaries^†^	Disease activity	3^q, x, y^
		Physical functioning	8^d, k, l, n, s, t, w, z^
	Outcomes^†^	Healthcare use	6^b, c, d, p, s, t^
		Mortality	2^b, c^
Humanistic	Intermediaries^†^	Coping capacity (including empowerment)^§^	13^b, c, i, k, I, m, p, q, r, t, v, y, z^
		Physiological measures	1^y^
		Social support	1^n^
		Disease activity	11^c, i, k, l, m, n, r, y, w, z, aa^
		Physical functioning	5^c, l, n, y, z^
		Emotional functioning	4^l, n, r, w^
	Outcomes^†^	Health and wellbeing	14^c, d, h, j, k, m, p, q, r, s, v, w, y, z^
		Return to work	1^c^
		Satisfaction (including patient/consumer satisfaction)^§^	2^c, t^
		Performance measures	1^m^
Other	Treatment modifiers^†^	Knowledge	1^a^
		Communication (including communication skills and interactions)^§^	4^a, e, f, g^
		Process evaluation	5^h, k, o, t, w^
		PCC performance measures (including documentation, care atmosphere and goal attainment)^§^	9^f, g, h, m, o, p, t, x, aa^
	Unintended outcomes^¶^	Identified unintended outcomes within: disease activity, communication, healthcare use and mortality.	4^e, i, k, t^

* For study reference letters, see Table 1.

^†^ Categories according to the ECHO model (for economic, clinical and humanistic outcomes).^24^

^‡^ Such evaluations include presenting direct costs and sometimes indirect costs.

^§^ As listed in a report by the Health Foundation.^25^

^¶^ Unintended/unanticipated outcomes/consequences/events as opposed to intended (treatment) benefits.^52^

## 4 DISCUSSION

A unifying theme among the 27 intervention studies was that they reported having integrated all or some of the cornerstones included in the gPCC model, that is, initiating, working and safeguarding the partnership between patients and healthcare professionals.^9^ Thus, regardless of care context or study population, the care was planned and follow‐ups were performed in collaboration and agreement with the patient.

The studies explored a wide variety of alternative study designs, contexts and outcome measures. Interventions in hospital‐based settings were overrepresented but interventions were also readily accessible in the patient's home, the community and in primary care. A positive effect of this diversity is that the usefulness of PCC has been evaluated in different healthcare contexts. The downside, however, is that this diversity results in difficulties when comparing results across studies, which may potentially impede adopting PCC in clinical practice. Several studies were conducted as multi‐centre studies, which is a methodological strength. Moreover, that half of the studies included a co‐design with patients in the development of the intervention acknowledges a basis in patients’ preferences and needs. However, a co‐design with patients should be mandatory in all clinical studies, especially in interventions based on person‐centred principles.

Six of the studies reported theory development within the research group or doing previous qualitative studies to guide the intervention, which further strengthens the potential that the intervention includes components in congruence with meeting the patients’ needs and individual characteristics. The theoretical frameworks used in the 27 interventional studies ranged from philosophy to theory and models, including Ricoeur,^26^ Smith,^27^ McCormack, McCance^28^ and Ekman et al.^9^ A theoretical framework can have varying levels of abstraction of which philosophy most often represents ontological and ethical assumptions on what a human being is and should be, frequently coupled with theories and models describing applications to different contexts.^29^, ^30^ However, the operationalization of ethics based on person‐centredness provided according to the gPCC model^6^ has been used in several studies without adding any other specific theory or model and has shown positive effects compared with controls.^31^, ^32^, ^33^ This way of concrete ethical guidance in research may be feasible when actions of change in a healthcare practice must be developed and tested and theories and models instead risk frustrating possibilities for exploration and openness for local and contextual adaptations. An additional international example is Wheat et al,^34^ who used the gPCC model^6^ as a frame of reference in their analysis of how health professionals enhance PCC in primary care in England. All 27 of the studies reported using the theoretical framework during the development of the intervention and 21 reported using more than one theory. Guidelines typically stress the importance of theory in the development of complex interventions.^16^, ^18^, ^23^ The use of different theories within one intervention could also be necessary because a few single theories can describe complex human behaviour and one intervention could include processes on different levels.^35^ Thus, one theory may be appropriate for understanding processes of change at the individual level but inappropriate at an organizational or societal level.^23^

The description of implementation strategies before and during the intervention reveals the complexity and efforts that need to be addressed in integrating a theoretical framework into clinical practice. The complexity in operationalizing a PCC intervention is also apparent in the combination of multiple actors involved in PCC. The complexity entails challenges on many levels from the preparation of healthcare professionals and the environment in the care setting, expertise in framework and doing PCC and long‐term financial foundations for sustainable design, evaluation and implementation. The complexity also acknowledges the need for future studies on implementation processes designed explicitly for PCC. A synthesis of the early research performed by the GPCC has been led by a team of researchers in England who concluded that the research provides a base of evidence for an ethically based, yet practical, framework for PCC in various clinical areas.^14^, ^15^, ^36^, ^37^, ^38^ Two of the studies explored experiences from researchers in seven projects who were conducting and implementing diverse interventions during this first period of the centre's existence.^14^, ^36^ Findings showed that structures in clinical practice (eg time, a specific clinical culture, systems for documentation, workload and a focus on delivering information) constrained implementation of the PCC. Interventions had to be adapted to the particular setting to implement the narrative partnership and documentation. However, a firm belief in the integrity of the PCC approach, ongoing education and competent professional providers facilitated the shift from conventional care to PCC. A successful implementation requires continued dialogue and close collaboration between researchers, patients and staff.^14^, ^36^

Most of the 27 studies included outcome measures from more than one dimension (ie economic, humanistic and clinical measures), indicating the complexity in measuring the effects of PCC interventions. Very few PIs reported a structured collection of unintended outcomes during the interventions. This lack of reporting could potentially be the result of adverse effects not expected by these types of intervention, as no changes were suggested in medical treatment. This reasoning agrees with previous findings that only 1 of 19 interventional studies of personalized care planning reported any harms of the interventions.^39^ Moreover, there was a focus on self‐reported/patient‐reported outcome measures (PROMs, humanistic intermediaries: self‐reported outcomes that indicate disease status but no hard endpoints) rather than measures registered by professionals. Focusing on self‐reported measures/PROMs may be the result that underscores the experience of the patient as a person, which is congruent with the ethical basis of person‐centredness.

However, the degree of evidence for the effects of a certain intervention may vary with group and context. Several studies have shown that PCC targets vulnerable groups, such as the most elderly,^40^ patients with low education^41^ and those admitted for acute inpatient care.^42^ In addition, qualitative evaluations can help explore for whom and under what circumstances an intervention may be most indicated. Such analyses were common among the 27 studies, which provides a more thorough knowledge of the context and processes involved in the implementation of the intervention. Some studies mainly focused on or included only treatment modifiers, indicating a focus on the processes and implementation strategies rather than on the evaluation of the PCC effects. Thus, some studies, regardless of their aim to improve the healthcare experience, focused on measuring outcomes among healthcare professionals. De Silva^25^ listed 120 person‐centred outcome measures, but most of these were seldom adopted in the studies conducted by researchers affiliated to the GPCC. This lack of apparent consensus in outcome measures can also be the reason for the 120 PCC outcome measures listed by De Silva.^25^ It could also account for the extensive database^43^ of measures for person‐centred coordinated care provided by the research group in South West England that evaluated PCC in primary care.^44^ In addition, some studies reported unexpected changes in the implementation of the intervention (such as unforeseen difficulties in recruiting participants). This issue may also be a consequence that PCC is a relatively new field of research. Indeed, several of the PIs reported that they viewed their interventional studies as pilot studies or to mainly provide methodological development rather than evaluating intervention effects.

The main strength of this study, which permitted the use of follow‐up questions, was the direct contact with the PIs conducting the 27 interventional studies included in the survey. Another strength was that the PIs could comment on preliminary results of the analysis. Still, the results were mainly based on the responses to the questionnaire. No original publications from the included projects were retrieved or analysed. Thus, some studies may have been categorized incorrectly because of misunderstanding or misinterpretation of the PIs’ descriptions or the lack of precision in the questions. For instance, no conclusion could be drawn on the specialist competence of different providers of the interventions. Although the questionnaire was constructed in accordance with recognized reporting and pilot tested, it was evident that some respondents interpreted some questions about the implementation of the interventions differently. In addition, several other PCC interventional studies initiated from other sources are being performed within GPCC, but which are not included in this paper.

### 4.1 Impact

The analysis of the 27 interventional studies in this paper indicates a need to prioritize research with comprehensive coverage of healthcare systems and not limit it to evaluating PCC within a single condition. In the present overview of GPCC‐funded studies, interventions in primary care were less common in contrast to previous reviews in which primary care was well represented.^33^, ^39^ However, one of the studies in the present investigation covered both hospital and primary care, which is highly uncommon in the international literature.^39^ Moreover, future studies should be designed with comparability to previous research in mind regarding the choices of outcome measures and with the ability to identify clinically relevant differences between groups. However, based on the present findings, together with those from other projects such as the collaborative action for person‐centred coordinated care initiative,^45^ the introduction of PCC into the healthcare systems needs to be carefully followed and evaluated to identify effective practices. A priority‐driven research agenda has been suggested and may be useful to the GPCC to support healthcare decision‐making while using resources effectively.^46^

Based on the findings on the apparent lack of consensus on outcome measures, even within a specific research centre, and even more so internationally, a recommended action is to develop a core outcome set for evaluating PCC, including not only economic, clinical and humanistic outcomes but also unintended outcomes, which are seldom reported in the identified PCC studies. Core outcome sets (ie an agreed standardized collection of outcomes) for clinical trials have been developed over the past years to ensure a minimum level of reported outcomes.^47^, ^48^ Such core outcome sets should be further developed in collaboration with patients, next‐of‐kin/carers^48^ and professional caregivers to ensure that PROMs and patient‐important outcomes^49^ are captured. Concerning PCC, it is also relevant to evaluate patient‐reported experience measures, that is, a measure of patients’ perceptions and observations on aspects of healthcare and healthcare services. Additional useful materials for such development work are available in a recently published paper from the WE CARE project, defining key aspects and enablers of developing their PCC‐based ‘Health Labs’.^50^

## 5 CONCLUSION

The theoretical frameworks used in the 27 interventional studies were consistent with the established ethical basis of PCC. There was a large variety of designs and intervention characteristics, which is indicative of the different contextual conditions and complexity of interventions in each study. In addition, outcome measures varied widely across studies. Consensus discussions among researchers in the field, nationally and internationally, are needed to ensure that comparisons between studies are feasible and accurate.

## CONFLICT OF INTEREST

The authors declare no conflict of interest.

## AUTHOR CONTRIBUTIONS

All authors contributed to the design and implementation of the research. IE acquired the funding. HG, IB, EJU and SJ performed the analyses and drafted the manuscript, and all authors discussed results and contributed to the final manuscript.

## Data Availability

All data relevant for this publication are available in the manuscript. The underlying survey responses are in Swedish. For queries about the survey or survey responses, please contact the corresponding author Hanna Gyllensten, at: hanna.gyllensten@gu.se.

## Appendix A The questionnaire

**Table:**

Content	Item
Background	Provide the name and status of the project (planning phase, ongoing or closed)
Description of intervention	Does the intervention contain any of the following cornerstones of PCC? Patient Narrative, Partnership, Shared decision‐making, Documentation, None of the above
	Describe other aspects of the intervention that makes it person‐centred
Development of intervention	Describe if and how theory guided the intervention (eg in content and selecting endpoints, etc)
	Describe if and how the development of the intervention was influenced by the setting in which the intervention should be evaluated (eg in relation to content or study population)
Pilot study	Was a pilot study performed to evaluate the feasibility of the study?
	If yes, describe the pilot study (eg study population, setting)
	If yes, describe the result of the pilot study and how it affected the final intervention
Intervention	Describe the study population
	Describe any materials used in the intervention, such as diaries, applications and written information
	Describe the intervention (procedures, activities or processes within the intervention)
	Describe the number of sessions when the intervention was provided for each respondent, such as time period, number of sessions, how often and length of sessions. When applicable, describe intensity and dose
	Describe what has been adapted in the intervention, tailored or titrated for each respondent. Describe how
	Were all planned procedures, activities or processes performed to the extent to which they were planned?
	If this were not the case, please describe.
	Describe those who provided the intervention (eg nurse, psychologist, etc) and if they underwent any specific education before the start of the intervention
	Describe where the intervention was performed
Evaluation	Describe the design of the study
	Describe inclusion and exclusion criteria
	Describe how the recruitment of respondents was performed
	Describe how the sample size was calculated
	Was randomization performed?
	If yes, how was randomization performed?
Endpoints	Describe primary and secondary endpoints (including health economy)
	Describe data collection (included variables and time points)
	Describe the aim and method for any qualitative evaluation of the intervention (eg interviews)
	Describe any unintended outcome of the intervention (both positive and negative)
	Describe the generalization of the results and transferability to other clinical settings
Implementation	Was the intervention implemented in clinical practice?
	If yes, describe the implementation
	Was the intervention implemented in other clinical settings than where the intervention was evaluated?
	If yes, describe the clinical setting
Publications	Attach publications from the interventional study
	Specify any planned publications from the interventional study
